# Aberrant polyploidization pathways in megakaryocytes as therapeutic targets in leukemia

**DOI:** 10.1097/MS9.0000000000004891

**Published:** 2026-04-21

**Authors:** Emmanuel Ifeanyi Obeagu

**Affiliations:** a Department of Biomedical and Laboratory Science, Africa University, Mutare, Zimbabwe; b Department of Molecular Medicine and Haematology, Faculty of Health Sciences, University of the Witwatersrand, Johannesburg, South Africa

**Keywords:** cell cycle dysregulation, endomitosis, leukemia, megakaryocyte polyploidization, therapeutic targets

## Abstract

Megakaryocyte polyploidization is a specialized process essential for platelet production, orchestrated by tightly regulated transcriptional programs, cell-cycle control, and signaling pathways. Disruption of this process – characterized by incomplete endomitosis, low ploidy, and maturation arrest – contributes to leukemic transformation, particularly in acute megakaryoblastic leukemia and related myeloid malignancies. Aberrant polyploidization arises from transcription factor dysregulation, cell-cycle abnormalities, hyperactive proliferative signaling, microenvironmental alterations, and genomic instability. These defects collectively promote proliferation, differentiation arrest, and malignant expansion of megakaryocytic progenitors. Therapeutic strategies targeting these pathways, including modulation of transcription factors, restoration of endomitotic progression, inhibition of JAK–STAT and RAS/MAPK signaling, epigenetic reprogramming, and niche-directed interventions, offer promising avenues to suppress leukemic growth and restore normal maturation. Understanding aberrant polyploidization as a driver of leukemogenesis provides a framework for developing precision therapies in megakaryocytic leukemia.

## Introduction

Megakaryocytes (MKs) are among the largest and most specialized hematopoietic cells, uniquely equipped to produce platelets through a complex process of maturation. Central to this process is polyploidization, achieved via endomitosis – a modified cell cycle in which DNA replication occurs without subsequent cytokinesis. This generates high-ploidy cells, often ranging from 8 N to 64 N or higher, providing the cytoplasmic volume, organelles, and transcriptional capacity necessary for extensive granule synthesis and proplatelet formation. Polyploidization is therefore not simply a developmental curiosity but a physiological imperative that ensures sufficient platelet output to maintain hemostasis. The process is tightly regulated by transcription factors such as GATA1, RUNX1, FLI1, and NF-E2, cell-cycle machinery including cyclins and cyclin-dependent kinases (CDKs), and signaling cascades such as the thrombopoietin (TPO)/MPL/JAK2 pathway. Interactions with the bone marrow microenvironment further refine polyploidization, providing structural support, cytokine cues, and access to vascular niches essential for platelet release^[^1–4^]^.

HIGHLIGHTS

- Aberrant polyploidization in megakaryocytes drives leukemic progression.
- Dysregulated CDKs, Aurora kinases, RhoA, and p53 pathways contribute.
- Targeting these pathways offers novel therapeutic strategies.
- Restoration of normal polyploidy improves differentiation and reduces malignancy.
- Combination therapies may enhance efficacy and overcome resistance.

Disruptions in this tightly controlled maturation program have emerged as a critical mechanism driving leukemogenesis. Aberrant polyploidization – manifested as incomplete endomitosis, low-ploidy megakaryocytes, and arrested cytoplasmic maturation – creates a pool of immature, proliferative cells prone to genomic instability. These defects are particularly evident in acute megakaryoblastic leukemia (AMKL), myelodysplastic syndromes, and certain myeloproliferative neoplasms, where maturation-arrested megakaryoblasts accumulate and dominate the bone marrow. The resulting leukemic clones exhibit heightened proliferative capacity, evasion of differentiation, and an increased propensity for mutation accumulation, establishing a permissive environment for malignant transformation^[^5,6^]^.

At the molecular level, aberrant polyploidization arises from a combination of genetic and epigenetic alterations. Mutations in transcription factors, such as GATA1s or RUNX1, disrupt the regulation of genes required for endomitosis and cytoplasmic remodeling. Dysregulation of the cell cycle, including hyperactivation of cyclins/CDKs and abnormalities in Aurora kinase signaling, prevents proper polyploid progression. Simultaneously, hyperactive proliferative pathways such as JAK–STAT, RAS/MAPK, and PI3K/AKT sustain leukemic proliferation while blocking differentiation. Aberrant interactions with the bone marrow microenvironment, including altered stromal signaling and extracellular matrix remodeling, further reinforce this immature, leukemic phenotype. Epigenetic perturbations, including DNA methylation changes and chromatin remodeling defects, consolidate the differentiation block, creating a stable leukemic state^[^7^]^.

Recognizing aberrant polyploidization as a central driver of leukemogenesis has profound therapeutic implications. Traditional cytotoxic approaches, while partially effective, fail to address the underlying maturation defects. Emerging strategies aim to restore normal polyploidization, correct transcription factor and signaling abnormalities, modulate the marrow niche, and exploit vulnerabilities created by cell-cycle dysregulation. This paradigm shift toward mechanism-driven therapy offers the potential for more precise, effective, and durable interventions in megakaryocytic leukemias^[^8,9^]^. This review aims to provide a comprehensive overview of aberrant polyploidization in megakaryocytes, exploring the physiological pathways governing normal endomitosis, the molecular mechanisms leading to defective polyploidization in leukemia, and the emerging therapeutic strategies targeting these pathways. By integrating insights from cell biology, molecular genetics, and translational research, this review highlights the pivotal role of polyploidization as both a mechanistic cornerstone of leukemogenesis and a promising target for intervention.

## Aim

This review aims to provide a comprehensive overview of the mechanisms underlying aberrant polyploidization in megakaryocytes and to explore how these pathways contribute to leukemogenesis. It further seeks to examine emerging therapeutic strategies that target transcriptional, cell-cycle, signaling, and microenvironmental abnormalities, with the goal of restoring normal polyploidization, promoting megakaryocyte differentiation, and improving treatment outcomes in megakaryocytic leukemias.

### Methods

This narrative review was conducted through a comprehensive literature search to synthesize current knowledge on aberrant polyploidization in megakaryocytes and its therapeutic implications in leukemia. Relevant articles were identified using electronic databases, including PubMed, Scopus, Web of Science, and Google Scholar, covering publications from 2000 to 2025. Search terms included combinations of keywords such as “megakaryocyte polyploidization,” “endomitosis,” “aberrant polyploidy,” “acute megakaryoblastic leukemia,” “myeloproliferative neoplasms,” “cell cycle dysregulation,” and “therapeutic targets.”

Both original research studies and review articles were considered, with emphasis on molecular mechanisms, transcriptional regulation, signaling pathways, microenvironmental interactions, and therapeutic strategies. Studies focusing on experimental models, including *in vitro* human megakaryocyte cultures, animal models, and clinical data, were included to provide mechanistic and translational insights. Articles were screened for relevance, quality, and recency, and data were extracted and synthesized in a narrative format to highlight key concepts, mechanistic pathways, and emerging therapeutic approaches. No formal meta-analysis was conducted, as the aim of the review was to integrate mechanistic, preclinical, and clinical evidence into a coherent narrative addressing the role of aberrant polyploidization in leukemogenesis and potential targeted interventions.

### Physiological polyploidization pathways in megakaryocytes

Megakaryocyte polyploidization is a central feature of normal hematopoiesis, enabling the production of vast numbers of platelets necessary for hemostatic balance. This process is accomplished through endomitosis, a modified cell cycle in which DNA is replicated repeatedly without cytokinesis. Unlike conventional mitosis, endomitosis allows megakaryocytes to achieve DNA contents ranging from 8 N to 64 N or higher, resulting in a large cytoplasmic volume capable of supporting organelle biogenesis, granule synthesis, and proplatelet formation. The high-ploidy state is not merely structural but also functional, providing the transcriptional capacity needed for the production of thousands of platelets from a single megakaryocyte^[^1,3^]^. Polyploidization is tightly orchestrated through an interplay of transcriptional, signaling, and cytoskeletal pathways. Transcription factors such as GATA1, RUNX1, FLI1, and NF-E2 are pivotal in this process, regulating genes essential for cytoskeletal remodeling, granule biogenesis, and platelet-specific protein expression. These factors integrate proliferative cues with differentiation programs, ensuring that megakaryocytes undergo the sequential steps of DNA replication, cytoplasmic expansion, and eventual proplatelet formation. Their precise regulation ensures a balance between proliferation and terminal maturation, preventing premature cell division or defective platelet production^[^10,11^]^.

The cell-cycle machinery of megakaryocytes is uniquely adapted to support endomitosis. Cyclins such as cyclin D1 and cyclin E, along with their associated CDKs, promote repeated entry into S-phase, while mitotic cyclins such as cyclin B are downregulated to suppress conventional mitosis. In parallel, the RhoA–ROCK–myosin II pathway, which normally drives cytokinesis, is carefully modulated to allow controlled failure of cell division. This combination of repeated DNA replication and aborted cytokinesis enables progressive increases in ploidy while maintaining cellular integrity^[^6,12^]^. Signaling pathways play a complementary role in coordinating polyploidization. Thrombopoietin (TPO), the principal cytokine regulating megakaryocyte development, binds to its receptor MPL, activating downstream JAK2–STAT pathways that promote survival, growth, and cytoplasmic maturation. Additional pathways, including RAS/MAPK and PI3K/AKT, provide proliferative and metabolic support, ensuring that the megakaryocyte can sustain multiple rounds of endomitosis while accumulating the resources necessary for platelet production^[^13,14^]^.

The bone marrow microenvironment further refines megakaryocyte maturation. Stromal cells, endothelial interactions, and extracellular matrix components such as collagen and fibronectin provide physical and biochemical cues that guide cytoskeletal remodeling and support proplatelet extension. Close proximity to sinusoidal endothelium facilitates the final stages of platelet shedding, highlighting the importance of niche interactions in successful polyploidization^[^15,16^]^. Cytoplasmic remodeling is intimately linked to the polyploid state. As DNA content increases, the transcriptional output of the cell rises, driving the synthesis of α-granules, dense granules, and membrane structures. These adaptations culminate in the formation of the demarcation membrane system and proplatelets, which are extended into marrow sinusoids for platelet release. High ploidy is therefore both a structural requirement and a functional enabler of efficient platelet biogenesis^[^17,18^]^.

### Aberrant polyploidization in leukemia

Aberrant polyploidization in megakaryocytes represents a central pathogenic mechanism in the development of leukemic disorders, particularly acute megakaryoblastic leukemia (AMKL) and related myeloid malignancies. In normal hematopoiesis, megakaryocytes undergo carefully orchestrated endomitosis, achieving high ploidy and terminal maturation before producing platelets. When this process is disrupted, however, megakaryocytes fail to attain sufficient ploidy, remain in an immature state, and retain proliferative capacity, thereby creating a fertile ground for leukemic transformation^[^19,20^]^. At the molecular level, this aberrancy arises from multiple, often overlapping, defects. Dysregulation of key transcription factors such as GATA1, RUNX1, FLI1, and NF-E2 impairs the genetic programs that coordinate DNA replication, cytoplasmic expansion, and proplatelet formation. For example, in Down syndrome-associated AMKL, somatic mutations produce a truncated GATA1s isoform that fails to drive endomitotic progression, resulting in low-ploidy megakaryoblasts with heightened proliferative potential. Similarly, mutations in RUNX1 compromise lineage specification and maturation, reinforcing the accumulation of immature, leukemic cells^[^11,21^]^.

Cell-cycle disturbances further exacerbate polyploidization defects. Hyperactivation of cyclins and cyclin-dependent kinases (CDKs) drives repeated S-phase entry, while dysregulation of mitotic cyclins and Aurora kinases prevents the normal completion of endomitosis. The RhoA–ROCK–myosin II pathway, which controls cytokinesis, may also be aberrantly modulated, leading to incomplete or abortive divisions. Collectively, these disturbances trap megakaryocytes in a proliferative, low-ploidy state, enhancing genomic instability and susceptibility to additional oncogenic mutations^[^22,23^]^. Signaling pathways contribute to the maintenance of this malignant phenotype. Hyperactivation of TPO/MPL/JAK2, RAS/MAPK, and PI3K/AKT pathways sustains proliferation, promotes survival, and inhibits differentiation, while microenvironmental interactions – such as altered stromal signaling, extracellular matrix remodeling, and disrupted vascular niche localization – further impede normal maturation. Leukemic megakaryocytes can also reshape the marrow niche, secreting cytokines that reinforce a supportive environment for malignant expansion while suppressing healthy hematopoiesis^[^24,25^]^. Aberrant polyploidization also promotes genomic instability, a hallmark of leukemia. Low-ploidy megakaryoblasts that fail to exit the cell cycle accumulate DNA damage, centrosome abnormalities, and chromosomal mis-segregation, creating aneuploid cells prone to malignant transformation. Epigenetic alterations, including DNA methylation changes and chromatin remodeling defects, further consolidate the differentiation block, stabilizing the leukemic state and enhancing resistance to conventional therapies (Table 1) ^[^26,27^]^.

**Table 1:** Aberrant polyploidization in leukemia.

Mechanism	Effect on megakaryocytes	Contribution to leukemogenesis
Transcription Factor Dysregulation (GATA1, RUNX1, FLI1, NF-E2)	Impaired endomitosis, reduced ploidy, maturation arrest	Promotes accumulation of immature, proliferative megakaryoblasts prone to leukemic transformation
Cell-Cycle Abnormalities (Cyclins/CDKs, Aurora kinases, RhoA–ROCK pathway)	Abortive mitosis, incomplete cytokinesis, replication stress	Generates low-ploidy cells with genomic instability, increasing mutation burden
Hyperactive Signaling Pathways (TPO/MPL/JAK2, RAS/MAPK, PI3K/AKT)	Sustained proliferation, blocked differentiation	Supports survival and expansion of malignant clones
Microenvironmental Alterations (Stromal remodeling, ECM disruption, vascular niche perturbation)	Impaired maturation cues, altered adhesion, niche hijacking	Provides a supportive environment for leukemic megakaryocytes and suppresses normal hematopoiesis
Genomic and Epigenetic Instability (DNA damage, aneuploidy, chromatin remodeling defects)	Disrupted DNA replication and chromatin structure	Facilitates accumulation of oncogenic mutations, stabilizing leukemic phenotype

### Therapeutic targeting of aberrant polyploidization pathways

Understanding the molecular basis of aberrant polyploidization in megakaryocytes has unveiled a range of therapeutic opportunities in leukemias characterized by megakaryocytic involvement, particularly AMKL and myeloproliferative neoplasms. By targeting the pathways that disrupt normal endomitosis, differentiation, and cytoplasmic maturation, therapies can both suppress leukemic proliferation and restore physiological megakaryocyte function^[^19,28^]^. One of the most promising strategies focuses on modulation of transcription factors that regulate polyploidization. Dysregulated factors such as GATA1, RUNX1, and FLI1 impede maturation and promote leukemic expansion. Small molecules, epigenetic modifiers, or gene-editing approaches aimed at restoring the activity of these transcription factors have the potential to reinstate endomitotic progression, increase ploidy, and drive leukemic blasts toward terminal differentiation. Epigenetic therapies, including histone deacetylase inhibitors (HDACi) and DNA methyltransferase inhibitors, can also correct transcriptional repression of polyploidy-related genes, alleviating differentiation blocks in malignant megakaryocytes^[^29–31^]^.

Another major therapeutic avenue involves targeting aberrant cell-cycle and mitotic machinery. Leukemic megakaryocytes often exhibit hyperactive cyclins, cyclin-dependent kinases (CDKs), and Aurora kinases, which disrupt normal endomitosis and maintain proliferative potential. Pharmacological inhibitors of these proteins can selectively impair proliferating leukemic MKs, reduce genomic instability, and promote terminal differentiation. In addition, agents that stabilize endomitotic progression or modulate cytokinesis may restore physiological polyploidization and reduce leukemic burden^[^32,33^]^. Signal transduction pathways represent a complementary target class. Hyperactivation of TPO/MPL/JAK2, RAS/MAPK, and PI3K/AKT pathways sustains proliferation and survival in leukemic megakaryocytes while blocking maturation. Targeted inhibitors, such as JAK inhibitors (e.g., ruxolitinib) or MEK/PI3K pathway inhibitors, can reduce leukemic cell proliferation, restore sensitivity to differentiation signals, and potentially synergize with transcriptional or cell-cycle modulators to correct polyploidization defects^[^24,34^]^.

The bone marrow microenvironment also plays a crucial role in supporting leukemic MKs, making it a compelling therapeutic target. Leukemic megakaryocytes remodel stromal cells, secrete cytokines, and disrupt extracellular matrix interactions to maintain an environment conducive to malignant expansion. Therapies that normalize niche interactions – such as CXCL12/CXCR4 antagonists or antifibrotic agents – can restore normal maturation cues, limit leukemic proliferation, and enhance the efficacy of other interventions targeting intrinsic MK defects^[^35,36^].^ Emerging immunotherapies offer innovative strategies for targeting aberrant megakaryocytes. Surface markers such as CD41, CD42b, and CLEC12A, which are differentially expressed on leukemic MKs, provide opportunities for monoclonal antibody therapies or CAR-T cell approaches. When combined with differentiation-promoting or cell-cycle-targeted therapies, these immune-based strategies can selectively eliminate malignant cells while sparing normal hematopoiesis^[^37–40^]^.

## Conclusion

Aberrant polyploidization in megakaryocytes represents a pivotal mechanism in leukemogenesis, linking transcriptional dysregulation, cell-cycle disturbances, signaling abnormalities, and microenvironmental alterations to malignant transformation. Failure to achieve high ploidy and complete cytoplasmic maturation traps megakaryocytes in a proliferative, immature state, promoting genomic instability and creating a cellular foundation for leukemia. Recognition of these processes has shifted the perspective from viewing defective polyploidization as a secondary phenomenon to appreciating it as a central driver of disease.

Therapeutic strategies targeting aberrant polyploidization pathways offer promising opportunities to restore differentiation, suppress leukemic proliferation, and improve clinical outcomes. Approaches that modulate transcription factors, correct cell-cycle abnormalities, inhibit hyperactive signaling pathways, normalize the bone marrow niche, and harness immunotherapeutic interventions collectively provide a mechanistic framework for precision treatment in megakaryocytic leukemias. Continued research into the molecular and epigenetic underpinnings of polyploidization is essential to translate these insights into effective, durable therapies. By targeting the root causes of aberrant megakaryocyte behavior, it is possible to envision interventions that not only treat leukemia but also restore normal hematopoietic function.
